# Acceptability and adoption of handheld computer data collection for public health research in China: a case study

**DOI:** 10.1186/1472-6947-13-68

**Published:** 2013-06-26

**Authors:** Xia Wan, H Fisher Raymond, Tiancai Wen, Ding Ding, Qian Wang, Sanghyuk S Shin, Gonghuan Yang, Wanxing Chai, Peng Zhang, Thomas E Novotny

**Affiliations:** 1 Institute of Basic Medical Sciences at Chinese Academy of Medical Sciences / School of Basic Medicine, Peking Union Medical College, Beijing, China; 2 San Francisco Department of Public Health, San Francisco, CA, USA; 3 University of California, San Francisco, San Francisco, CA, USA; 4 Institute of Basic Research and Clinic Medicine, China Academy of Chinese Medical Sciences, Beijing, China; 5 Sydney School of Public Health, University of Sydney, Camperdown, New South Wales, Australia; 6 Graduate School of Public Health, San Diego State University, San Diego, CA, USA

**Keywords:** Surveys, Electronic data collection, Handheld computers, China

## Abstract

**Background:**

Handheld computers for data collection (HCDC) and management have become increasingly common in health research. However, current knowledge about the use of HCDC in health research in China is very limited. In this study, we administered a survey to a hard-to-reach population in China using HCDC and assessed the acceptability and adoption of HCDC in China.

**Methods:**

Handheld computers operating Windows Mobile and Questionnaire Development Studio (QDS) software (Nova Research Company) were used for this survey. Questions on tobacco use and susceptibility were drawn from the Global Adult Tobacco Survey (GATS) and other validated instruments, and these were programmed in Chinese characters by local staff. We conducted a half-day training session for survey supervisors and a three-day training session for 20 interviewers and 9 supervisors. After the training, all trainees completed a self-assessment of their skill level using HCDC. The main study was implemented in fall 2010 in 10 sites, with data managed centrally in Beijing. Study interviewers completed a post-survey evaluation questionnaire on the acceptability and utility of HCDC in survey research.

**Results:**

Twenty-nine trainees completed post-training surveys, and 20 interviewers completed post-data collection questionnaires. After training, more than 90% felt confident about their ability to collect survey data using HCDC, to transfer study data from a handheld computer to a laptop, and to encrypt the survey data file. After data collection, 80% of the interviewers thought data collection and management were easy and 60% of staff felt confident they could solve problems they might encounter. Overall, after data collection, nearly 70% of interviewers reported that they would prefer to use handheld computers for future surveys. More than half (55%) felt the HCDC was a particularly useful data collection tool for studies conducted in China.

**Conclusions:**

We successfully conducted a health-related survey using HCDC. Using handheld computers for data collection was a feasible, acceptable, and preferred method by Chinese interviewers. Despite minor technical issues that occurred during data collection, HCDC is a promising methodology to be used in survey-based research in China.

## Background

Over the last 20 years, handheld computers have been commonly used in medical care settings for data collection and retrieval [1,2]. Such applications include e-prescribing, ordering and checking laboratory tests, billing, and medical record-keeping [1]. Reported examples of handheld computer data collection (HCDC) include monitoring treatment for headache [3], pain research [4], and research on bipolar disorders [5]. Outside medical care settings, HCDC is increasingly used for population surveys, such as for HIV risk behavior [6] and for tobacco use [7]. The use of HCDC is particularly helpful in field settings where it is inconvenient or inappropriate to use bulky, paper-based surveys or desktop/laptop computers. Such settings might include entertainment venues (bars and nightclubs), homes, and remote areas in developing countries [8-10]. In addition, HCDC data management is more accurate, reliable, and timely compared to paper-and-pencil data collection and manual data entry, and it has been well-accepted by users of such technology [11,12]. HCDC has been commonly used for research in developed countries, and it is increasingly used in the developing world for field data collection [13-15].

In China, HCDC use in health-related research is still rare. A PubMed search using the terms “China” and “handheld data collection” returned no results. Furthermore, an informal search using a number of major Chinese city names in place of “China” produced only two studies, both pertaining to medical care settings only. In 2008, the Chinese Center for Disease Control and Prevention (CCDC) used HCDC for mortality surveillance after the Wenchuan earthquake. In that setting, there was limited Internet access for reporting mortality data to the CCDC, and HCDC proved to be valuable in this field setting. In 2009, the CCDC also used HCDC for the Global Adult Tobacco Survey (GATS), conducted in 100 counties/districts in China [16]. However, in that study, an English-language data entry structure was developed by US researchers rather than by Chinese collaborators, and thus, Chinese investigators were not able to fully participate in data management and analyses during the study.

In order to improve health survey research methods in China, local and provincial health personnel should be able to independently conduct surveys using HCDC, including the development of data collection and management systems for handheld computers. This paper describes a developmental project using a HCDC for a cross-sectional tobacco-use survey among female rural-to-urban migrant workers in China. We report evaluation results based on post-training and post-data collection surveys of interviewers who completed the study. The results of this study may encourage broader use of HCDC, particularly with difficult-to reach populations.

## Methods

### The field data collection system

For this study, we used handheld computers (Hewlett-Packard iPaqs, 212) running Windows Mobile (version 5) and Questionnaire Development Studio (QDS) software (version 2.6, Nova Research, Bethesda, Maryland, USA).The handheld computer featured handwriting recognition software which allows direct input of Chinese characters by interviewers. The QDS software uses a versatile method of transferring questionnaires onto the handheld computer. The construction of the questionnaire for the device involves formulating a question prompt, a variable type, and an explanatory note for each question. The variable types were defined as numeric, logical, date, or character, and could be validated by range checks on numeric and date fields, code validity on single-coded fields, branch conditions, or consistency checks against previously recorded data. If required, records could be automatically date-, time- and identity-stamped, and access to the system can be protected by passwords. Several questionnaires can be uploaded to one handheld computer at the same time. When a questionnaire is selected for data entry, several functions can be used simultaneously, such as editing, optional help, built-in error-message displays, data summary, and record review. QDS’ design environment also provides the option to build electronic surveys for a variety of hardware platforms including handheld and newer tablet computers. This software also has a data management feature (QDS Warehouse), which allows data transfers to Excel, SPSS, or other software formats. QDS (version 2.6) supports Unicode characters, allowing questions, responses and instructions to be displayed as Chinese characters. Collected data can also be stored as Chinese characters. While other software (e.g., Entry ware, Pen dragon Forms) provides many of these features, including Unicode characters, QDS was chosen for a combination of reasons, such as ease of use in a visual programming environment, cost, and applicability across hardware platforms (e.g., handheld, tablet, laptop and desktop computers).

### Study procedures

The main study was designed to assess susceptibility to smoking initiation among young rural-to-urban migrant women who were working as restaurant or hotel workers or as commercial sex workers. The protocol used in this study was approved by the Institutional Review Boards at Peking Union Medical College and San Diego State University. Study interviewers administered the survey after obtaining verbal informed consents from eligible participants. Verbal permissions were recorded by the interviewers on the intake screens of the HCDC. Details regarding the sampling, procedures, survey instrument and institutional review have been previously reported [17]. Briefly, a total of 2229 rural-to-urban migrant women (1697 restaurant and hotel workers aged 18–24 years and 532 commercial sex workers aged 18–30 years) were enrolled in ten provincial capital cities. For restaurant and hotel workers, surveys were conducted in local CCDC offices where they visited for a mandatory annual health screening. For commercial sex workers, data were collected in entertainment venues where they worked and periodically received health education.

### Programming

**Figure 1:**
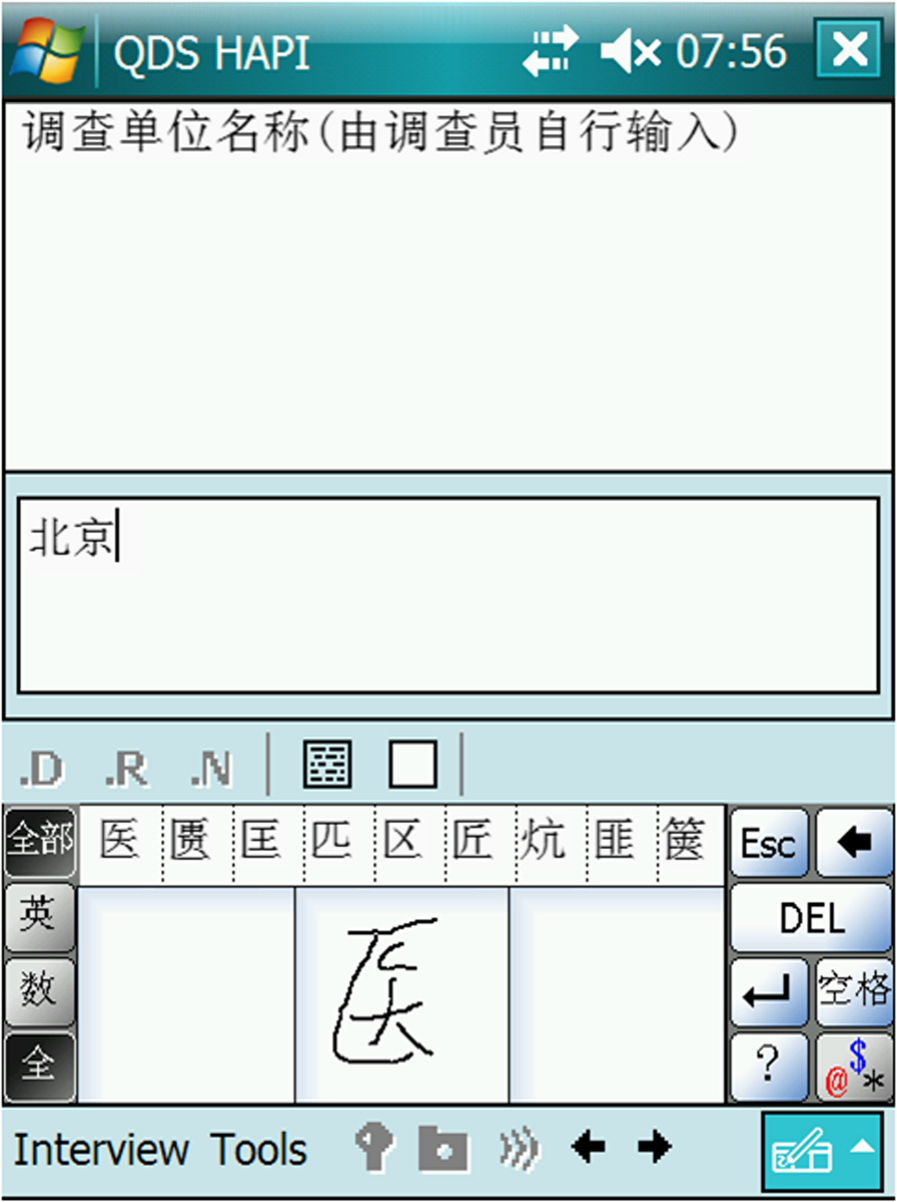
Screen Capture, Chinese-language data collection instrument, China, 2010.

After receiving a half-day training and technical assistance by a US-based investigator (co-author SS) on developing the data entry screens, constructing variables, and data transfer, a China-based information technology specialist (co-author TCW) programmed the electronic survey (Figure 1). Selected variables from a 100-question survey used by CCDC in an ongoing study were defined (Table 1).

**Table 1:** Broad structure of the questionnaire used in the study, China, 2010

**Field type**	**No. of questions**	**Contents**
Character	65	smoking environment and attitudes; smoking and anti-smoking advertisement; cigarette marketing; knowledge of health risks; and attitudes towards anti-smoking policies
Logical	12	smoking status
Open-ended	9	other specified information
Numerical	12	socioeconomic and demographic characteristics; general health behaviors; smoking-related behaviors;
Date and time	2	start and end times of interviews; age of interviewers

### Pilot study

After completing the initial programming, a pilot study was conducted in two cities (Beijing and Hohhot) in April 2010. During the pilot study, investigators administered surveys to a convenience sample of migrant women from local CCDC health examination centers and entertainment venues. Any problems or issues identified during this pilot study were documented and addressed by CCDC investigators. The data transfer process using QDS warehouse was also pilot-tested at both sites.

### Interviewer training

A three-day HCDC training session was conducted in July 2010 in Beijing. Two interviewers and one supervisor from each of the 10 participating provinces attended the training (n = 29, with one supervisor absent). The training protocol was developed collaboratively by the US and Chinese investigators. The content included an overview of the main study’s goals, recruitment techniques, an orientation of the functions of the handheld computers, a review of the electronic data collection instrument, and instructions for data entry, transfer, and management. Each interviewer had an opportunity to practice using HCDC during the training.

### HCDC field data collection

Data from study participants were collected after verbal informed consent was recorded on the HCDC. The questions included different data entry types (e.g., pop-up lists, one-option answers). Interviewers entered data using a pen stylus and specified entry options. Interviewers were required to select a response before moving on to the next question. The program also allowed interviewers to return to previous questions within the same interview to modify answers if necessary.

### Data management

Data were managed with the QDS Warehouse system, which aggregates collected data into one dataset and can be used to export datasets to standard statistical packages. Due to limited resources, only one warehouse system was installed at the data-coordinating center in Beijing. At the end of each data collection day, interviewers in each city transferred handheld computer data to a laptop computer, encrypted the data, and emailed the data file to the data-coordinating center. The coordinating center then combined records from all project sites through the QDS warehouse system. The software automatically deleted duplicate records, located incomplete records, and converted data into an Excel, SPSS, or other formats for data analysis. Finally, the Beijing coordinating center provided feedback to the senders to verify complete transmission of data at the end of each data collection day.

### Evaluations

The evaluation included three components: 1) an anonymous post-training survey administered to all trainees (interviewers and supervisors), 2) a quality-control evaluation by supervisors, and 3) an anonymous post-data collection survey of interviewers. We did not conduct a pre-training survey because all interviewers and supervisors stated that they had no previous experience with HCDC. The post-training questionnaire was administered at the end of interviewer training. It included 16 questions about the self-assessment of skills, knowledge, and acceptability regarding HCDC. The quality-control evaluation component involved a supervisor at each study site observing interviewers conducting interviews and completing a 23-item questionnaire about the interviewing process. Each supervisor observed 10 interviews. Finally, after completing the main field study, all interviewers completed an 18-item evaluation questionnaire via email; this addressed recruitment, data collection, and the overall experience using HCDC.

## Results

### Pilot study findings

During the pilot study, 150 women from Beijing and 136 women from Hohhot were recruited and interviewed. The pilot study revealed that it was overly time-consuming to read the full consent form on a small screen; that there were erroneous skip patterns, wording, and terminology problems; that the operational instructions appeared only in English; and that entering Chinese characters using the handwriting recognizer was too time-consuming (Table 2).

**Table 2:** Problems and solutions identified during pilot testing of a hand-held computer data collection system, China 2010

**Issue**	**Solution adopted**
Time consuming to read the full consent form on a small screen	Development of a short version to show on the handheld computer screen and a printed full version for interviewees to read and keep
Erroneous skip patterns, logical problems, wording and terminology problems	On-going revision of the questionnaire and programming in the HCDC system prior to final survey administration
Operational instructions appeared only in English	Development of a paper reference guide that provides Chinese translations for commonly used commands
Entering Chinese characters using the handwriting recognizer was time-consuming	Continuous practice to improve data entry (Time required to complete surveys changed from 25–30 minutes to 15–17 minutes after intensive practice).

### Field survey findings

During the larger field survey, interviewers encountered problems such as the HCDC system freezing and having SD card-reading errors; these problems were usually solved by rebooting the system. In addition, some interviewers reported persistent difficulty in entering Chinese characters with the stylus into the handheld computer. These technical problems impeded communication between the interviewers and interviewees. However, most interviewees were comfortable with the HCDC, and only two potential interviewees refused to participate due to fears that the HCDC device would breach their confidentiality.

### Evaluation findings

#### Interviewers’ attitude and skills using HCDC

Twenty interviewers and nine supervisors completed training (n = 29 trainees). Among the 11 female and 9 male trainee interviewers, the mean age was 31 years and almost all (90%) had tertiary education. Immediately after the training, 28 of 29 trainees felt confident about their ability to collect survey data using the handheld computer, 27 felt confident about transferring data, 26 felt confident about encrypting data files, and 24 felt confident that they could solve the problems they might encounter while using HCDC. Nineteen would recommend the use of HCDC for future surveys.

#### HCDC vs. Paper-and-pencil survey methods

After the field data collection was completed, 16 of 20 interviewers thought that HCDC operations were very easy or easy and that they felt confident about their ability to use the handheld device, and 12 felt they could solve problems on HCDC that arose during the survey activity (Table 3). Overall, 19 out of all 29 trainees (15 of 20 interviewers) thought the handheld device was more efficient in data collection, whereas three thought the paper-and-pencil method was more efficient and two had no preference. Sixteen trainees reported that they would prefer to use the HCDC system in future projects, while five would prefer the paper-and-pencil method. Nineteen trainees overall would recommend the use of handheld data collection in future surveys. The two interviewers who thought paper-based methods more efficient and would prefer using paper-based methods in the future were female and relatively older compared to other trainees (43 and 45 years of age compared to the overall average age of 31 years). More than half (n = 11) of the interviewers thought that the handheld device was a particularly useful method for collecting field data in China.

**Table 3:** Post-training trainees self-assessment (n = 29) & post-data collection interviewer assessment (n = 20), China, 2010

		**1**		**2**		**3**		**4**		**5**		**9**	
		**N**	**%**	**N**	**%**	**N**	**%**	**N**	**%**	**N**	**%**	**N**	**%**
Post-training trainees self-assessment (n = 29)	I feel confident about my ability to ask survey questions.^#^	13	44.8	16	55.2	0	0	0	0	0	0	0	0
	I feel confident about how to record the response of the interviewees. ^#^	12	41.4	17	58.6	0	0	0	0	0	0	0	0
	I feel confident about my ability to collect survey data using the handheld device. ^#^	15	51.7	13	44.8	0	0	1	3.45	0	0	0	0
	I feel confident about my ability to transfer study data from the handheld device to a laptop or a desktop. ^#^	15	51.7	12	41.4	1	3.5	0	0	0	0	1	3.5
	I feel confident in my ability to compress and password protect data files.^#^	15	51.7	11	37.9	1	3.5	0	0	0	0	2	6.9
	I feel confident that I can solve many of the problems that I might face while using the handheld. ^#^	9	31.0	15	51.7	2	6.9	1	3.5	0	0	2	6.9
	I feel confident that I will receive help to solve problems that I might face and I can’t solve while using the handheld. ^#^	9	31.0	20	69.0	0	0	0	0	0	0	0	0
	I would recommend the use of handheld data collection in survey work in the future. ^#^	10	34.5	9	31.0	5	17.2	1	3.5	0	0	4	13.8
post-data collection interviewer assessment (n = 20)	How would you rate your experience collecting data using handheld device? ^##^	3	15.0	13	65.0	2	10.0	2	10.0	0	0	0	0
	How would you rate your experience transferring data using handheld device? ^##^	8	40.0	8	40.0	3	15.0	0	0.0	1	5.0	0	0
	How would you rate your experience encrypting data using handheld device? ^##^	10	50.0	8	40.0	1	5.0	0	0.0	1	5.0	0	0
	After this project, I feel confident about my ability to collect survey data using the handheld device. ^#^	6	30.0	10	50.0	2	10.0	2	10.0	0	0	0	0
	I feel confident about solving problems that I might face while using the handheld^#^	1	5.0	11	55.0	1	5.0	4	20.0	0	0	3	15.0

^#^:1 = Strongly agree; 2 = Agree; 3 = Neither agree nor disagree; 4 = Disagree; 5 = Strongly disagree; 9 = Not sure.

^##^: 1 = Very easy; 2 = Easy; 3 = Not easy, not difficult; 4 = Difficult; 5 = Very difficult; 9 = Not sure.

#### Quality control

Results from 203 completed quality-control evaluations by supervisors showed that all the interviewers used the HCDC correctly. During the preparation process, 201 of 203 interviews were evaluated as “fully” prepared with all necessary materials. During eligibility screening, in all but one interview, the interviewer could “fully” orient participants and could successfully enter participants’ occupation, age, and hukou status to determine eligibility. In all observed interviews, the interviewers offered participants a copy of the consent form and sufficiently explained the study to the interviewees. During questionnaire administration, all could ‘usually’ or ‘fully’ do well in reading questions exactly as written, definitions verbatim, and all response options exactly as written , as well as recognizing inconsistent responses, clarifying responses, and correcting data entry in HCDC. Based on supervisors’ observation and assessment, the pace for 11% of the interviews was ill-timed (“too slow”, “too fast” or “much too fast”), and 6% of observed interviews were “too long” or “far too long”. All “usually” or “always” used all of the appropriate flashcards, anchored time periods with examples, and used non-leading probes. During all but two interviews, interviewers “usually” or “always” probed incomplete and inappropriate responses. All but one established good rapport with participants, including making eye contact and maintaining a neutral attitude, conveyed by verbal or non-verbal behavior.

#### Interviewer recommendations

Interviewers provided the following suggestions for improving HCDC programming in future studies:

1) Features can be included to improve the user-friendliness of the system, such as a progress bar that shows both interviewers’ and interviewees’ progress;

2) To improve time efficiency, some information should be stored in the handheld device between interviews. For instance, the interviewers’ information and the interview site do not need to be entered anew for each interview;

3) The numerical response scale was separate from null answers such as ‘don’t know’ and ‘refuse to answer’ in the same question. Additionally, the null responses were only available in English, which led to confusion among the interviewers.

## Discussion

Mobile technology has become increasingly useful in public health research and interventions. Compared to the paper-and-pencil method for data collection, handheld devices can provide range and consistency checks at the time of the interview, guide interviews with predefined routing, error and feedback messages, and randomly select interviewees as part of household surveys [15,18]. Furthermore, HCDC can rapidly provide data in an analysis-ready format, thereby eliminating the labor-intensive and error-prone process of data entry. A systematic review compared HCDC and paper-and-pencil data collection used in randomized controlled trials and found HCDC to be more time-efficient and better for data management [12]. Furthermore, HCDC can be particularly useful in surveys involving sensitive data such as sexuality, drug use, and other risk behavior [13]. Although limitations were identified by some users, evaluation results from this study suggest that using handheld computers is an acceptable, feasible, and preferred method for data collection in health research in China.

China now has more than 1.37 billion people [19] and is facing many challenges in public health. Population surveys are important in the surveillance of health behaviors and in measuring intervention outcomes in China. Most previous behavioral surveys in China used paper-based questionnaires and manual data entry. This laborious approach created excessive burdens for investigators, given the large sample sizes often required in such research. Based on the feasibility and acceptability of HCDC demonstrated by the present study, it is reasonable to conclude that HCDC can be an effective and efficient data collection method for population health research in China. Furthermore, as newer hardware platforms (e.g., tablet computers) become more widely used for mobile computing, similar software platforms (such as QDS) can be used on these devices.

There were significant advances in the use of HCDC in the current project compared to previous projects using HCDC in China. First, the current study used QDS software for programming, which is more user-friendly for designing questionnaires in handheld computers compared to the previous system. Second, the current project emphasized capacity building. Using a “train the trainers” model, the Chinese technical and supervisory staffs were able to develop the questionnaire and complete the programming with minimal assistance from US technical experts. When problems occurred, the Chinese technical experts could then provide immediate technical guidance and trouble-shooting to interviewers and other staff. Finally, we encountered fewer technical difficulties compared to the previous GATS project that used HCDC. Qualitative evaluation of that project suggested that some interviewers found it difficult to master handheld devices due to low computer proficiency. In the current study, almost all interviewers had a tertiary degree and thus great computer proficiency that allowed them to acquire new skills in HCDC. This implies that computer proficiency might be an important selection criterion for interviewers in studies using HCDC.

This study also revealed some limitations and areas of improvement for HCDC. The major technical limitation we found was the difficulty of QDS programming in a non-English language. QDS’ underlying structure and programming environment are only available in English. For example, as mentioned above, response options on some questions, such as “not applicable” or “unknown” cannot be displayed in Chinese. In order to resolve this problem, investigators developed detailed instructions for interviewers on how to use the English response items. However, updates in the QDS software for use in non-English language settings are needed. These improvements will require collaborative efforts among investigators, interviewers, programmers, and software designers.

There were also limitations with the evaluations we conducted. For example, we did not include a pre-training assessment because no interviewer reported having experience with HCDC. Some data such as on attitudes towards using HCDC could have been collected before training to compare with post-training data. During the post-training assessment, we did not collect demographic information on the interviewers and supervisors as these surveys were to be anonymous. In addition, the quality control evaluation might have introduced bias because the interviewers might have modified their behavior while being directly observed. Nevertheless, this study shows acceptability and feasibility of HCDC in the Chinese public health research environment. With advances in software development and even more user-friendly mobile devices, such data collection methodologies should supplant most paper and pencil data collection methods.

## Conclusions

Despite the current software limitations, HCDC is a promising approach for data collection in public health research in China. Future studies using HCDC in international settings should focus on capacity building of local staff and software development. A “train the trainer” model should be used in such projects. With repeated use, trainees will likely prefer use of handheld or other mobile device data collection systems. In addition to survey data collection, therapeutic applications using mobile technology are now becoming more common [20], thus opening significant new pathways for technologically-driven public health practice and research.

## Competing interests

The authors declare that they have no competing interests.

## Authors’ contributions

XW designed, implemented the study, contributed to the writing and final editing of the paper. HFR provided training in the use of handheld technology and data management systems, drafted, revised and edited the final paper. TCW developed the Chinese language survey, supervised data management, evaluated the implementation, drafted, revised and edited the final paper. DD evaluated the implementation, co-drafted the first draft and provided critical revision and edits of the final paper. QW evaluated the implementation, drafted, revised and edited the final paper. SS evaluated the implementation, drafted, revised and edited the final paper. GHY evaluated the implementation, drafted, revised and edited the final paper. WXC evaluated the implementation, drafted, revised and edited the final paper. PZ evaluated the implementation, drafted, revised and edited the final paper. TEN provided overall direction and supervision to all aspects of the project, drafted, revised and edited the final paper. All authors read and approved the final manuscript.

## Pre-publication history

The pre-publication history for this paper can be accessed here:

http://www.biomedcentral.com/1472-6947/13/68/prepub
